# Complete Genome Sequence of GD1108, a Moderate-Virulence Strain of Human-Associated ST398 Methicillin-Susceptible Staphylococcus aureus

**DOI:** 10.1128/MRA.00687-19

**Published:** 2019-07-11

**Authors:** Jo-Ann McClure, Steven M. Shideler, Kunyan Zhang

**Affiliations:** a Centre for Antimicrobial Resistance, Alberta Health Services/Alberta Public Laboratories/University of Calgary, Calgary, Alberta, Canada; b Department of Pathology & Laboratory Medicine, University of Calgary, Calgary, Alberta, Canada; c Department of Microbiology, Immunology and Infectious Diseases, University of Calgary, Calgary, Alberta, Canada; d Department of Medicine, University of Calgary, Calgary, Alberta, Canada; e The Calvin, Phoebe and Joan Snyder Institute for Chronic Diseases, University of Calgary, Calgary, Alberta, Canada; Georgia Institute of Technology

## Abstract

Staphylococcus aureus multilocus sequence type 398 (ST398) is responsible for an increasing number of severe infections in humans. There are no reports detailing if all ST398 strains are equally virulent. We present the genome sequence of the moderate-virulence ST398 methicillin-susceptible Staphylococcus aureus strain GD1108, determined in a Caenorhabditis elegans infection model, to reveal the ST398 sublineage virulence.

## ANNOUNCEMENT

Staphylococcus aureus multilocus sequence type 398 (ST398) was first reported as an animal pathogen; however, it gained notoriety when it was found associated with human diseases, ranging from minor localized infections to more severe invasive illnesses (1–17). In humans, there appears to be augmented pathogenicity of ST398 infection (2, 8, 18), but no studies existed comparing the relative virulence of the different sublineages. A preliminary analysis from testing a collection of ST398 isolates using the Caenorhabditis elegans infection model revealed three lineages with high, moderate, or low virulence and mean nematode killing rates of 90%, 67%, and 44%, respectively. Whole-genome sequencing was done on representatives from each group, with the goal of detecting genetic determinants that could be responsible for the differing levels of toxicity between the strains. In separate reports, we presented the full chromosomal sequences of the high-virulence strain GD487 and the low-virulence strain GD1696. The complete genome sequence of the moderate-virulence strain GD1108 is presented here.

Strain GD1108 was isolated from a school child from a prevalence survey in 2011 in Guangzhou, People’s Republic of China. The genome was generated from the hybrid assembly of PacBio and Illumina sequencing reads. Phenol-chloroform extraction was used to isolate genomic DNA from an overnight bacterial culture, started from a single colony of GD1108 in brain heart infusion broth and grown at 37°C. Illumina library preparation and sequencing were done at the Centre for Health Genomics and Informatics at the University of Calgary, Canada, using the recommended conditions. The library was prepared using the NEBNext Ultra II FS DNA fragment library kit, and then 600 cycle MiSeq v3 sequencing was done. A sheared large-insert PacBio library was prepared at the Genome Quebec Innovation Centre in Montreal, Canada, using Covaris g-TUBEs and the SMRTbell template prep kit 1.0. Sequencing of the library with PacBio RS II sequencing technology, using one single-molecule real-time (SMRT) cell, was also performed at Genome Quebec. Raw Illumina reads had adapters trimmed and sequences with quality scores of <20 removed using Cutadapt v1.15, while sequence quality was assessed with FastQC v0.11.5 (19) (http://www.bioinformatics.babraham.ac.uk/projects/fastqc). Filtered PacBio subreads (prepared by Genome Quebec) and trimmed Illumina reads were used for hybrid genome assembly using the Unicycler v0.4.7 pipeline (SPAdes v3.13.0, minimap, Racon v1.3.2, Pilon v.1.23) (20–24). Once assembled, GC content was determined with QUAST v4.4, and gene annotation was accomplished using NCBI’s Prokaryotic Genome Annotation Pipeline using the best-placed reference protein set (GeneMarkS-2+ v4.8) (25, 26). All programs were run using their default settings.

Following assembly, three contigs were generated from the GD1108 reads, including one representing the chromosome and two representing plasmids (3,175 and 18,638 bp). From PacBio sequencing, there were 1,516,326,066 sequenced bases covered by 110,591 raw reads, with an average read length of 13,711 bp. The average read lengths for Illumina sequencing were 261 bp for R1 and 262 bp for R2, with 412,567 reads generated. The estimated genome coverage for Illumina sequencing was 39×, while PacBio sequencing had 487× coverage. The assembled GD1108 chromosome was 2,783,012 bp long, with 2,808 genes identified, of which 2,727 were coding DNA sequences (CDS), 81 were RNA genes, and 84 were pseudogenes. The GC content was 32.98%.

### Data availability.

The chromosomal genome sequence was deposited at GenBank under the accession number CP040230, with SRA accession numbers SRX5915513 (Illumina) and SRX5915514 (PacBio).
